# The Clinical Effects of Aromatherapy Massage on Reducing Pain for the Cancer Patients: Meta-Analysis of Randomized Controlled Trials

**DOI:** 10.1155/2016/9147974

**Published:** 2016-01-14

**Authors:** Ting-Hao Chen, Tao-Hsin Tung, Pei-Shih Chen, Shu-Hui Wang, Chuang-Min Chao, Nan-Hsing Hsiung, Ching-Chi Chi

**Affiliations:** ^1^Department of Public Health, Kaohsiung Medical University, Kaohsiung 807, Taiwan; ^2^Department of Medical Research and Education, Cheng Hsin General Hospital, Taipei 112, Taiwan; ^3^Faculty of Public Health, School of Medicine, Fu Jen Catholic University, Taipei 242, Taiwan; ^4^Department of Dermatology, Far Eastern Memorial Hospital, New Taipei 220, Taiwan; ^5^Department of Business Management, National Taipei University of Technology, Taipei 106, Taiwan; ^6^College of Management, National Taipei University of Technology, Taipei 106, Taiwan; ^7^Department of Dermatology, Chang Gung Memorial Hospital, Chiayi 613, Taiwan; ^8^College of Medicine, Chang Gung University, Taoyuan 333, Taiwan

## Abstract

*Purpose*. Aromatherapy massage is an alternative treatment in reducing the pain of the cancer patients. This study was to investigate whether aromatherapy massage could improve the pain of the cancer patients.* Methods*. We searched PubMed and Cochrane Library for relevant randomized controlled trials without language limitations between 1 January 1990 and 31 July 2015 with a priori defined inclusion and exclusion criteria. The search terms included aromatherapy, essential oil, pain, ache, cancer, tumor, and carcinoma. There were 7 studies which met the selection criteria and 3 studies were eventually included among 63 eligible publications.* Results*. This meta-analysis included three randomized controlled trials with a total of 278 participants (135 participants in the massage with essential oil group and 143 participants in the control (usual care) group). Compared with the control group, the massage with essential oil group had nonsignificant effect on reducing the pain (standardized mean difference = 0.01; 95% CI [−0.23,0.24]).* Conclusion*. Aromatherapy massage does not appear to reduce pain of the cancer patients. Further rigorous studies should be conducted with more objective measures.

## 1. Introduction

Alternative therapies are frequently used to relieve various symptoms of patients. They are used instead of standard medical treatments and alternative therapies are distinct from complementary medicine which is meant to accompany, not to replace, standard medical practices. Alternative therapies can include various well-known treatments such as acupressure, acupuncture, massage, aromatherapy, diet, and herbal medicine [1]. Patients with cancer have many disturbing symptoms that influence their quality of life [2]. Almost all patients with cancer are affected by pain, insomnia, stress, and depression. These symptoms cause severe discomfort preventing the cancer patients from having the complete physiology and psychology to face the cancer and as a result, the treatment could not get the best.

Pain occurs in up to 70% of patients with advanced cancer and about 65% of patients dying of nonmalignant disease [3]. This symptom is a complex subjective phenomenon and is affected by the emotional context in which it is endured [4]. Cancer patients frequently express the desire to have open and honest dialogue with medical carers about pain. Pain relief is vital to the treatment of cancer. Despite the widespread use and recognition of clinical recommendations for the management of cancer-related pain, avoidable suffering is still prevalent in patients with malignant disease [5]. Alternative therapies have been commonly used in reducing the discomfort of the patients with cancer [6]. However, the effects of alternative therapies on the patients with cancer were still controversial.

Aromatherapy has been used in various diseases [7]. Aromatherapy is usually used in combination with massage; however, the effects of massage with essential oil on the different symptoms such as pain, stress, anxiety, depression, and insomnia were still controversial [8]. The objective of this study was to investigate the effects of massage with essential oil on reducing the pain of the patients with cancer.

## 2. Materials and Methods

### 2.1. Literature Search and Search Strategy

We searched the potential relevant publications between 1 January 1990 and 31 July 2015 on PubMed and Cochrane library; the search strategy was “(aromatherapy OR essential oil) AND (pain OR ache) AND (cancer OR tumor OR carcinoma)” without limitations for language.

### 2.2. Study Selection

Studies were included if they met the following inclusion criteria: (1) the study design was randomized controlled trial, (2) the subjects were human, (3) the experimental group received massage with essential oil and the control group received usual care only, and (4) mean difference and standard deviation were reported in the article. The components of essential oil and the spices of cancer were not discussed here. The title or abstract of all publications which were similar to the outcome was reviewed to evaluate whether to include them or not. The full texts were checked carefully to see if there was any potentially related information.

### 2.3. Data Extraction

The following data were extracted from included eligible studies through a data extraction form: first author, year of publication, country of publication, study period, assigned group, randomly assigned participants, types of participants, component of essential oil, intervention time, and methods used for assessing the intensity of pain. Furthermore, we used the Cochrane Collaboration tool to assess the risk of bias of the included trials and evaluated the following 7 domains associated with bias of intervention: random sequence generation, allocation concealment, blinding of participant and personnel, blinding of outcome assessment, incomplete outcome data (Attrition bias: it refers to systematic differences between groups in withdrawals from a study lead to incomplete outcome data. Exclusions refer to situations in which some subjects are omitted from reports of analyses, despite outcome data being available to the trial lists.), selective reporting, and other biases (bias due to problems not covered elsewhere).

### 2.4. Statistical Analysis

The Review Manager 5.3 (The Nordic Cochrane Centre, The Cochrane Collaboration, 2014) was used for meta-analysis. We presented the mean difference (MD) or standardized MD with 95% confidence interval (CI) for continuous data. Because the baseline which assesses the intensity of pain was different, we used standardized data to adjust the different baseline. Heterogeneity in meta-analysis refers to the variation in study outcomes between studies. In this study, we used *χ* ^2^ and *I* ^2^ inconsistency statistics. *I* ^2^ statistic describes the percentage of variation across studies which is due to heterogeneity rather than chance [12]. A *P* value of less than 0.10 indicated significant heterogeneity. *I* ^2^ values of 0% to 24.9%, 25% to 49.9%, 50% to 74%, and 75% to 100% were considered as none, low, moderate, and high heterogeneity. A 95% CI for *I* ^2^ is constructed using the iterative noncentral chi-squared distribution method [13]. In addition, we used the fixed-effect model when *I* ^2^ was less than 75% and would have used the random-effects model if *I* ^2^ had been 75% or more. For analyzing the continuous data, if the SD was not reported, we estimated SD by standardized mean difference and 95% CI.

## 3. Results

### 3.1. Literature Search and Studies Characteristics

**Figure 1:**
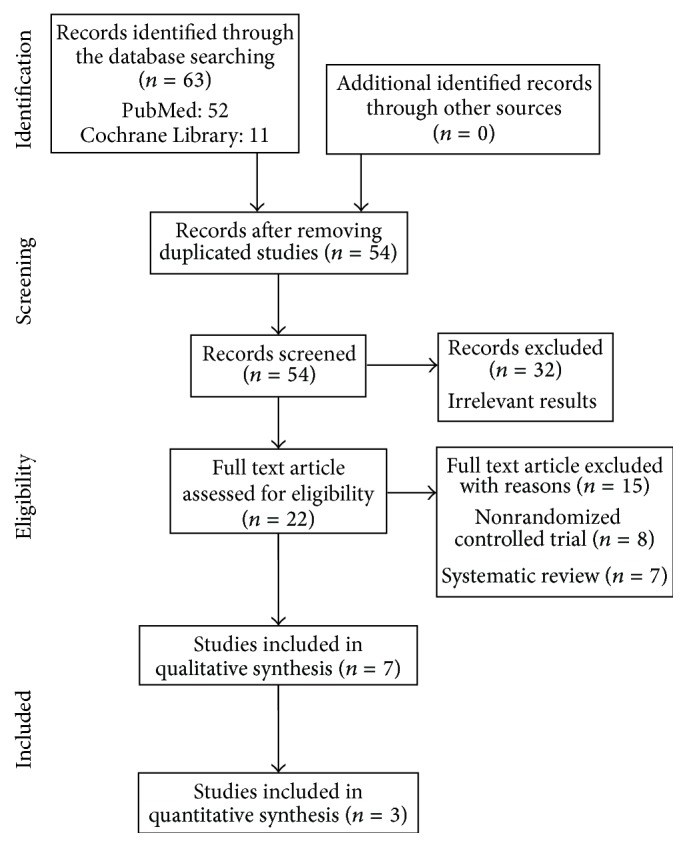
PRISMA (preferred reporting items for systematic reviews and meta-analyses) flow diagram.

Figure 1 showed the search process and the final selection of relevant trials by the preferred reporting items for systematic reviews and meta-analyses (PRISMA) guidelines [14]. We obtained 63 records from PubMed and Cochrane Library and further removed 9 duplicated studies and excluded 47 records that did not meet our inclusion criteria. Eventually, three randomized control trials (Wilkie et al., S. P. Weinrich and M. C. Weinrich, and Wilkinson et al.) with 278 participants were included in this systematic review and meta-analysis [9–11].

**Figure 2:**
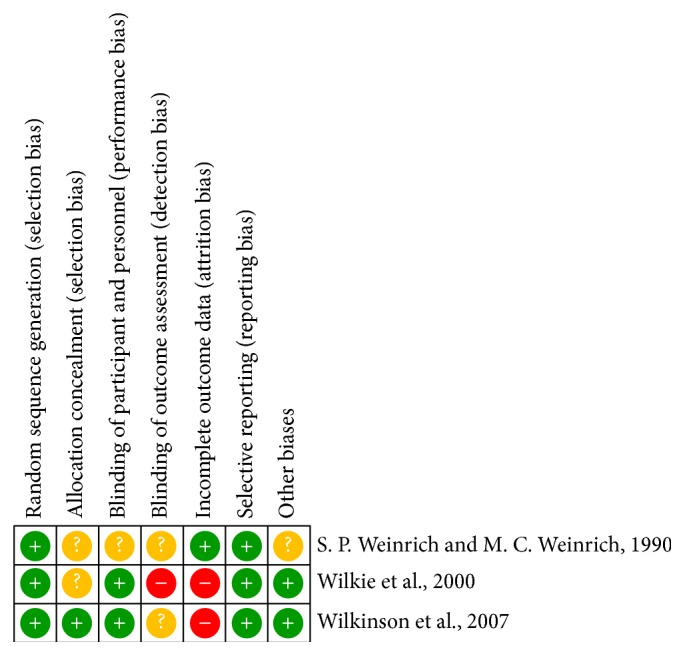
Risk of bias summary: authors' judgements about each risk of bias item for each included study.

The characteristics of the included trials are summarized in Table 1. These trials were published from 1990 to 2014. The sample size was from 14 to 115, with a total of 278 participants (143 participants in the control group and 135 participants in the massage with essential oil group). All three trials were not double-blinded. Two trials had a low risk of performance bias (Wilkie et al. and Wilkinson et al.). As to attrition bias, the two trials (Wilkie et al. and Wilkinson et al.) had a high risk of bias. The article of Wilkie et al. indicated that there were 55 participants in the beginning, but there were only 29 participants at the end of the study, with 15 in the massage group and 14 in the control group, respectively. And the article of Wilkinson et al. indicated that there were 288 participants in the beginning. However, 38 were lost to follow-up in the experimental group and 29 were lost to follow-up in the usual care group. As for other biases, two trials did not have other potential biases, so these two trials had no problem in the column of other biases (Wilkie et al. and Wilkinson et al.) However, in the trial of S. P. Weinrich and M. C. Weinrich, there was no explanation about the potential bias. All the included trials of risk were assessed by the Cochrane Collaboration's tool for assessing the risk of bias appraisal (Figure 2). In all trials, the participants of experimental group accepted massage with essential oil. However, the components of essential oil were not explained carefully.

### 3.2. The Effects on Reducing the Pain of the Patients with Cancer

**Figure 3:**
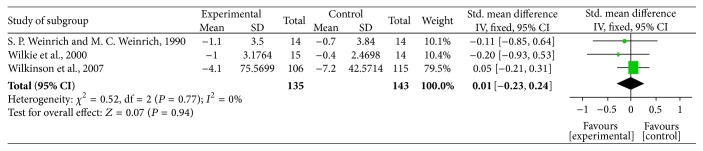
Meta-analysis based on the mean difference between massage with essential oil and usual care.

We pooled the data from the included trials using the fixed-effect model because of no heterogeneity (chi-square value = 0.52, *P* = 0.77, and *I* ^2^ = 0%) (Figure 3). The pooled standardized mean difference (SMD) was 0.01 (95% CI [−0.23,0.24]). And the test for overall effect obtained *P* = 0.94. There was no significant difference in pain reducing effect between massage with essential oil and usual care.

**Figure 4:**
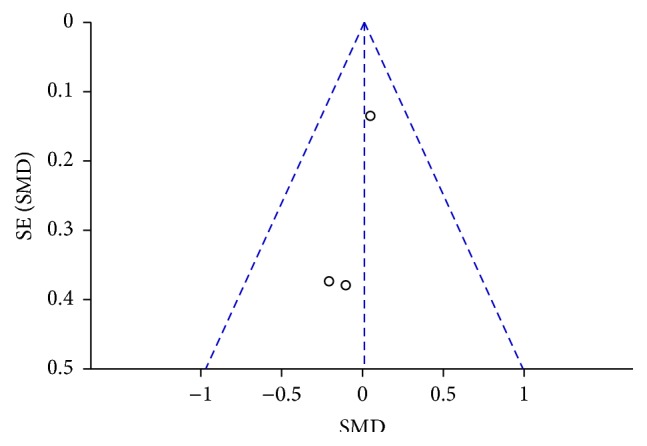
Funnel plot based on Wilkie et al., S. P. Weinrich and M. C. Weinrich, and Wilkinson et al.

Publication bias was defined as the publication or nonpublication of studies depending on the direction and statistical significance of the results, and the first systematic investigations of publication bias focused on this aspect of the problem. As Figure 4 shows, the funnel plot was symmetry, indicating no publication bias in this study.

## 4. Discussion

### 4.1. Clinical Implications

The popularity of complementary and alternative medicine (CAM) is growing among the general public, and, in many developed countries, its use varies from 70% to 80% [15]. No comprehensive systematic review has been published since 1998 about the frequency with which cancer patients use CAM [16]. One undated meta-analysis further indicated that the combined prevalence for “current use” of CAM across all studies was 40%. The highest prevalence was in the United States and the lowest was in Italy and Netherlands. Meta-analysis suggested an increase in CAM use from an estimated 25% in the 1970s and 1980s to more than 32% in the 1990s and to 49% after 2000 [17]. CAM use has been associated with sociodemographic factors because many studies have found that increased CAM use is associated with female gender, higher levels of education, and good income [15, 18]. In addition to associated demographic characteristics, the type of cancer and its clinical stage are also two important factors related to the use of CAM [15].

Aromatherapy encompasses the use of essential oils derived from different types of plant sources for a variety of application methods [19]. These oils can be absorbed into the body via the skin or the olfactory system [20]. The proponents of aromatherapy lay claim to an ancient tradition of herbal medicine practiced in countries such as Egypt and India thousands of years ago. However, the term was initially used by the French chemist Gattefossé in a book first published in 1936 [21]. Previous studies also indicated that, for cancer patients, claims of benefits include reduced anxiety levels and relief of emotional stress, pain, muscular tension, and fatigue [22]. However, trials of aromatherapy meet formidable methodological problems due to the fact that the smell of the oils is difficult to mask and patient blinding is therefore difficult.

Systematic reviews and meta-analysis aim to collate and synthesise all studies that meet prespecified eligibility criteria using methods that attempt to minimize bias [23]. Regardless of the extent of heterogeneity across studies, we still believe that all these studies are attempting to measure the same effect, even though with varying success. The varying success in estimating this is then a consequence of systematic and random error [24]. In this study, the meta-analysis included three randomized controlled trials that compared the outcomes of massage with essential oil and usual care. About the quality of three trails, we evaluate the risk of bias by the Cochrane Collaboration's tool (Figure 2); one of the trials needs to notice its quality (S. P. Weinrich and M. C. Weinrich); there are many questions about how to allocate the participants and application of blind. Nevertheless, this trial (S. P. Weinrich and M. C. Weinrich) is still an important evidence in the area of aromatherapy. In the statistical analysis, we found that there were no significant differences in pain reduction between the massage with essential oil and usual care groups. The possible reason was that the study designs of each research were not complete; that is, the outcomes of this meta-analysis were only to investigate the association between massage with essential oil and usual care, but they did not explore whether the essential oil should be added or not. Therefore, it is very difficult to fully prove whether the massage was clinically effective on reducing pain among the patients with cancer. For inconsistency and heterogeneity, Figure 4 shows that there was no publication bias and chi-square value = 0.52, *P* value = 0.77, and *I* ^2^ = 0% indicate that there was no heterogeneity in this study. In addition, based on the previous results, there is no published literature that provides a sound rationale for the use of aromatherapy massage as a medical intervention. It is probably best considered as a pleasant diversion for those who can afford it and are prepared to pay for it in the absence of hard efficacy data for lasting and relevant health effects [21].

From the methodological viewpoint, there were still several limitations in this meta-analysis. The major one was the fact that the amount of trials which could be searched was too insufficient and the statistical power could be lower due to smaller sample sizes. Nevertheless, the serious bias in this meta-analysis was publication bias; in the overall meta-analysis of randomized controlled trials there was no significant on the publication bias (test for overall effect: *Z* = 0.07, *P* = 0.94) (Figure 4), but the results might be influenced by low statistical power of insufficient studies [25]. Another bias in this study is the controversy surrounding random-effects models; that is, the assumption of normally distributed random effects violates the basic principle of randomization in statistical inference [26]. The hypothetical common variance of these so-called random effects would serve only as a nuisance variable if there were no random effects. The end result of the application of this nuisance variable to meta-analytic weights would then be to markedly increase estimator variance and equalize the weights through penalizing the larger studies [27, 28]. A further limitation is that the study lacked one more equivalent treatment control group to estimate the superior effectiveness of aromatherapy massage. Therefore, it is not clear whether the positive effects were due to the aromatherapy, the massage, or both.

## 5. Conclusions

In conclusion, our data did not suggest that aromatherapy massage may be effective in reducing pain for the cancer patients. We also cannot completely elucidate the nonspecific effects of aromatherapy. Further randomized studies should include more objective measures to explain the possible mechanism of reduction in pain due to cancer.

## Figures and Tables

**Table 1:** Characteristics of included randomized controlled trials.

Author	Publication year	Country	Study period	Assigned group	Randomly assigned participants (*n*)	Types of participants	Component of essential oil	Intervention time	Methods used for assessing pain intensity
Wilkinson et al. [9]	2007	UK	1998–2002	Control: usual care Experimental: massage with essential oil	115 116	Patients with any type of cancer	Unclear	10 weeks	EORTC (European Organisation for Research and Treatment of Cancer)

Wilkie et al. [10]	2000	USA	1995-1996	Control: usual care Experimental: massage with Essential oil	14 15	Patients with any type of cancer	Unclear	2 weeks	PAT (Pain Assessment Tool)

S. P. Weinrich and M. C. Weinrich [11]	1990	USA	Unclear	Control: no physical contact Experimental: massage with Essential oil	14 15	Patients with any type of cancer	Unclear	10 minutes	VAS (Visual Analogue Scale)
